# Nanofabrication Technologies to Control Cell and Tissue Function in Three-Dimension

**DOI:** 10.3390/gels9030203

**Published:** 2023-03-07

**Authors:** Hidenori Otsuka

**Affiliations:** Department of Applied Chemistry, Faculty of Science, Tokyo University of Science, 1-3 Kagurazaka, Shinjuku-ku, Tokyo 162-8601, Japan; h.otsuka@rs.tus.ac.jp; Tel.: +81-3-5228-8265; Fax: +81-3-5261-4631

**Keywords:** biointerface, 3D culture, micropatterning, PEGylation, cell array

## Abstract

In the 2000s, advances in cellular micropatterning using microfabrication contributed to the development of cell-based biosensors for the functional evaluation of newly synthesized drugs, resulting in a revolutionary evolution in drug screening. To this end, it is essential to utilize cell patterning to control the morphology of adherent cells and to understand contact and paracrine-mediated interactions between heterogeneous cells. This suggests that the regulation of the cellular environment by means of microfabricated synthetic surfaces is not only a valuable endeavor for basic research in biology and histology, but is also highly useful to engineer artificial cell scaffolds for tissue regeneration. This review particularly focuses on surface engineering techniques for the cellular micropatterning of three-dimensional (3D) spheroids. To establish cell microarrays, composed of a cell adhesive region surrounded by a cell non-adherent surface, it is quite important to control a protein-repellent surface in the micro-scale. Thus, this review is focused on the surface chemistries of the biologically inspired micropatterning of two-dimensional non-fouling characters. As cells are formed into spheroids, their survival, functions, and engraftment in the transplanted site are significantly improved compared to single-cell transplantation. To improve the therapeutic effect of cell spheroids even further, various biomaterials (e.g., fibers and hydrogels) have been developed for spheroid engineering. These biomaterials not only can control the overall spheroid formation (e.g., size, shape, aggregation speed, and degree of compaction), but also can regulate cell-to-cell and cell-to-matrix interactions in spheroids. These important approaches to cell engineering result in their applications to tissue regeneration, where the cell-biomaterial composite is injected into diseased area. This approach allows the operating surgeon to implant the cell and polymer combinations with minimum invasiveness. The polymers utilized in hydrogels are structurally similar to components of the extracellular matrix in vivo, and are considered biocompatible. This review will provide an overview of the critical design to make hydrogels when used as cell scaffolds for tissue engineering. In addition, the new strategy of injectable hydrogel will be discussed as future directions.

## 1. Introduction

Drug screening requires in vivo work. This is because in vivo experiments have an advantage over in vitro experiment, in that the entire biological response can be taken into account when determining the time-dependent response to a new drug candidate. However, in vivo toxicity testing using human bodies often presents problems. Therefore, the development of microscale cell culture systems, using microfabrication and cell culture technologies as in vitro human surrogates, will contribute to a safe and much-awaited screening method. Recent advances of cell culture at the microscale have made it possible to analyze and detect the function of newly synthesized drugs and unknown toxicants [1,2,3,4,5,6,7,8,9].

On the other hand, two-dimensional cell culture methods are the global standard, but definitely do not reflect physiological conditions, and, therefore, do not reproduce tissue anatomy or physiology useful for research or industrial applications. Performing a three-dimensional culture of cells is an appropriate idea for studying physiological cellular functions, but it requires a multidisciplinary approach and expertise. Three-dimensional (3D) culture requires consideration of the design of scaffolds that guide cell organization, and the introduction of bioreactors that control the exchange of oxygen, nutrients, and waste products. A 3D culture was established by several methods, including the use of biopolymer scaffolds, hydrogels, cell sheets, bioreactors with perfusion, microfluidics, and hanging drops. As 3D culture systems become more developed and able to mimic human and animal tissue function, the design and introduction of co-cultures will make stem cell utilization highly possible. Cell spheroids are also three-dimensional models that do not require cell scaffold material, they can be generated from a variety of cell types, and they are formed from adherent cells to aggregate via intercellular adhesion. Spheroids are usually produced by monoculture or co-culture techniques, and their cell morphologies and functions can be easily imaged by fluorescence or confocal laser scanning microscopy (CLSM). As a result, spheroids have been used for high-throughput screening, for example, in solid tumor growth model studies and drug discovery research. This review summarizes a general overview and the useful techniques for constructing 3D culture models. Particular focus is paid to surface engineering techniques, used in cellular micropatterning [10,11,12,13,14,15,16,17,18,19,20,21,22,23,24,25,26,27,28,29,30] as important tools to obtain spheroid micropatterns [31,32,33,34,35]. To develop 3D cell microarrays, provided by a cell’s non-adherent surface and a cell-adhesive region, it is important to microfabricate a protein-repellent surface, because cellular adhesion and proliferation are regulated by protein adsorption.

**Figure 1:**
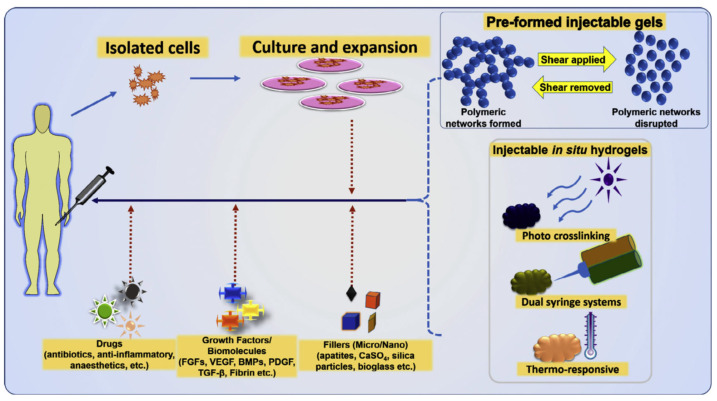
Schematic outlining pre-formed injectable hydrogels along with different in situ-formed hydrogels, and the possibilities of using them as carriers of cells, drugs, growth factor/biomolecules and fillers. Reprinted with permission from Ref. [1]. Copyright 2015, Elsevier.

Cell therapies offer a tailorable, personalized treatment for use in tissue engineering, to address defects arising from trauma, inefficient wound repair, or congenital malformation. However, most cell therapies have achieved limited success to date. Typically injected in solution as monodispersed cells, transplanted cells exhibit rapid cell death or insufficient retention at the site, thereby limiting their intended effects to only a few days. Spheroids, which are dense, three-dimensional (3D) aggregates of cells, enhance the beneficial effects of cell therapies by increasing and prolonging cell–cell and cell–matrix signaling. The use of spheroids is currently under investigation for many cell types. To improve the therapeutic effect of cell spheroids even further, various biomaterials (e.g., fibers and hydrogels) have been developed for spheroid engineering [36,37,38,39,40,41,42,43,44,45,46,47,48,49,50,51,52,53,54,55,56,57,58,59]. Note that hydrogels, as artificial 3D cell scaffolds, have attracted great attention in recent years because of their ability to reproduce the biological environment, with their inherent biochemical and physicochemical properties, such as cell adhesion, degradation, and viscoelasticity (Figure 1) [1]. Hydrogels can mimic the characteristics of the extracellular matrix (ECM) of many tissues, and various molecular designs have been proposed to tune the mechanical strength and degradation speed of hydrogels, resulting in significant advances in their biomedical applications. The cross-linking methods for forming networks consisting of biopolymers and synthetic polymers are described in detail, and are classified into (i) covalent cross-linking, (ii) dynamic covalent cross-linking, and (iii) physical crosslinking. Finally, recent progresses in molecularly improved biopolymer hydrogels and interpenetration network hydrogel formation will be presented for their applications as tissue scaffolds.

## 2. Cell-Patterning Techniques

Microfabrication techniques are used to form patterned cells, or spheroids, on cell culture substrates. This cell patterning is a necessary component of basic research in biosensors, tissue engineering, viral infection, and other areas that utilize the functional responses of cells. We have perfected dry-etching (or plasma-etching) techniques for creating micro-patterned cells. Photolithography technology, a commonly used semiconductor technology, is also very well-developed, and has been widely utilized in cell patterning. However, this photo-processing technology has disadvantages in applications for analyzing specific biological activities the biocompatibility of cells, due to cytotoxicity. Therefore, microcontact printing, microfluidic patterning using microfluidic channels, and laminar flow patterning have been developed one after another as alternative techniques to animal experiments, for the comprehensive analysis of biological functions. This paper outlines dry etching as a cell-patterning method that can be formed by a particularly simple procedure.

### 2.1. 2D Cell Patterning

Atypical cell–cell interactions between hepatic parenchymal and nonparenchymal cells have been reported to induce cell growth, migration, and differentiation. In vitro, co-cultures of hepatic parenchymal and nonparenchymal cells have been used to maintain and regulate the hepatocyte phenotype. Toner et al. [13]. have performed pioneering research in this field by utilizing microfabrication techniques, allowing for a more precise control of cell–cell interactions through “cellular patterning” or “micropatterning”.

Recent advances in microfabrication techniques have made possible new studies that allow us to freely control the role of interfaces, formed by heterogeneous cell populations and the ratio of cell populations, and have provided some new insights into the complex modes of intercellular communication in these co-cultures. In the future, improved microfabrication techniques will enable the development of a wide range of materials with spatial resolution in the nano-scale range, such as various biomolecules, as well as the tailoring of these cell array techniques to test specific biological hypotheses. The introduction of cocultures into bioreactors will be useful for the construction of bioreactors for clinical support. In addition, cell culture would be applied to both basic research in cell communication, organogenesis, and physiology, as well as in the development of functional tissue constructs for medical applications. Below is an overview of typical cellular micropatterning methods.

### 2.2. Dry Etching (Plasma Etching)

**Figure 2:**
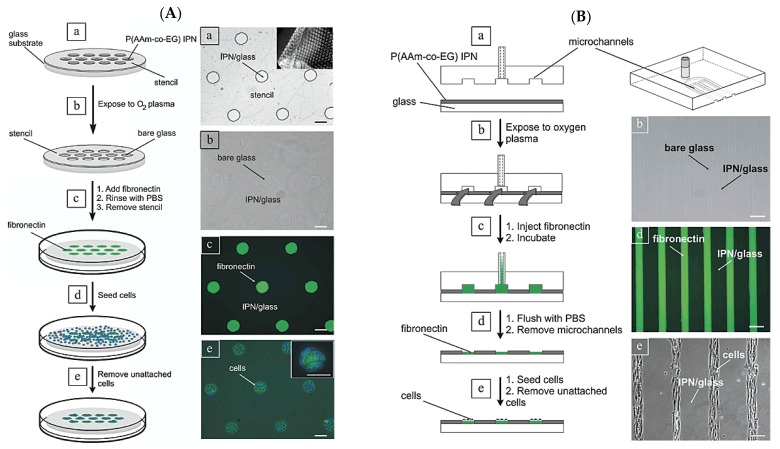
(**A**) Schematic illustration (left column) and demonstration (right column) of the procedure for cellular micropatterning based on the use of PDMS stencils. (**a**) The stencil is placed onto a glass substrate homogeneously grafted with the IPN. A picture of the stencil before (inset) and after application onto the surface is shown on the right. (**b**) The stencil serves as a mask for selective etching of the IPN in an oxygen plasma. The etching leaves islands of IPN-free glass surrounded by IPN (as shown in the corresponding phase-contrast image on the right). (**c**) Fluorescently tagged fibronectin (Fn) is adsorbed onto the plasma-etched islands of glass, and the stencil is removed. (**d**) The fibronectin/IPN-patterned substrate is incubated with a cell suspension. (**e**) Unattached cel ls are removed by exchanging the medium. The inset shows a fluorescence image of C2C12 myoblasts (blue) attached to the fibronectin islands (green). Scale bars are 100 μm. (**B**) Schematic illustration (left column) and demonstration (right column) of the procedure for cellular micropatterning based on the use of PDMS microchannels. (**a**) The PDMS mold is placed onto a glass substrate, homogeneously grafted with the IPN. (**b**) The IPN is selectively removed after exposure of the surface to oxygen plasma, which leaves lines of IPN-free glass surrounded by IPN (see phase-contrast image on the right). (**c**,**d**) Fluorescently tagged fibronectin is injected into the microchannel network. After flushing with PBS, the microchannels are removed, leaving lines of fibronectin surrounded by IPN (see fluorescence image of the adsorbed fibronectin on the right). (**e**) The fibronectin/IPN-patterned substrate is incubated with a cell suspension and unattached cells are removed by exchanging the medium. Shown on the right is a phase-contrast image of C2C12 myoblasts attached to the lines of fibronectin. Scale bars are 100 μm. Reprinted with permission from Ref. [13]. Copyright 2015, Elsevier.

Folch et al. [14] reported a new method for cell patterning at the micro-scale, using oxygen plasma. The technique (1) equably grafts glass substrates with an interpenetrating polymer network (IPN) [P(AAm-co-EG)] of poly(acrylamide) and poly(ethylene glycol) to create a protein-rejecting surface, and (2) uses oxygen plasma to remove this coating in a position-selective manner. They propose elastomeric stencils (i.e., self-sealing membranes with through-holes, Figure 2A) and microchannels (Figure 2B) as removable masks that allow for the selective removal of IPN regions unprotected by the mask by oxygen plasma etching. When the stencil or microchannels are removed after plasma etching, cell-adhesive regions appear, separated by the non-adhesive regions of cells.

### 2.3. Patterning of Spheroids as 3D-Microorganized Cells

The dry-etching technique we have studied involves the irradiation of ions (usually nitrogen, chlorine, or boron trichloride plasma) through a metal mask pattern to remove a portion of the material from the polymer surface to be patterned. Usually, dry etching is done in the linear or anisotropic direction, unlike many chemical-etching methods used in wet etching. By employing this dry etching, two-dimensional microarrays of 10,000 hepatocyte heterogeneous spheroids (100 × 100) underlaid with endothelial cells as feeder cells were precisely formed on microfabricated glass substrates, coated with polyethylene glycol (PEG) brushes at 100 μm intervals in a of 20 × 20 mm area [30]. The co-culture of hepatocytes and endothelial cells is essential for long-term hepatocyte viability and liver-specific function, yielding hepatocyte spheroids with 100 μm in diameter that function as miniaturized, albumin-secreting livers for at least 3 weeks.

**Figure 3:**
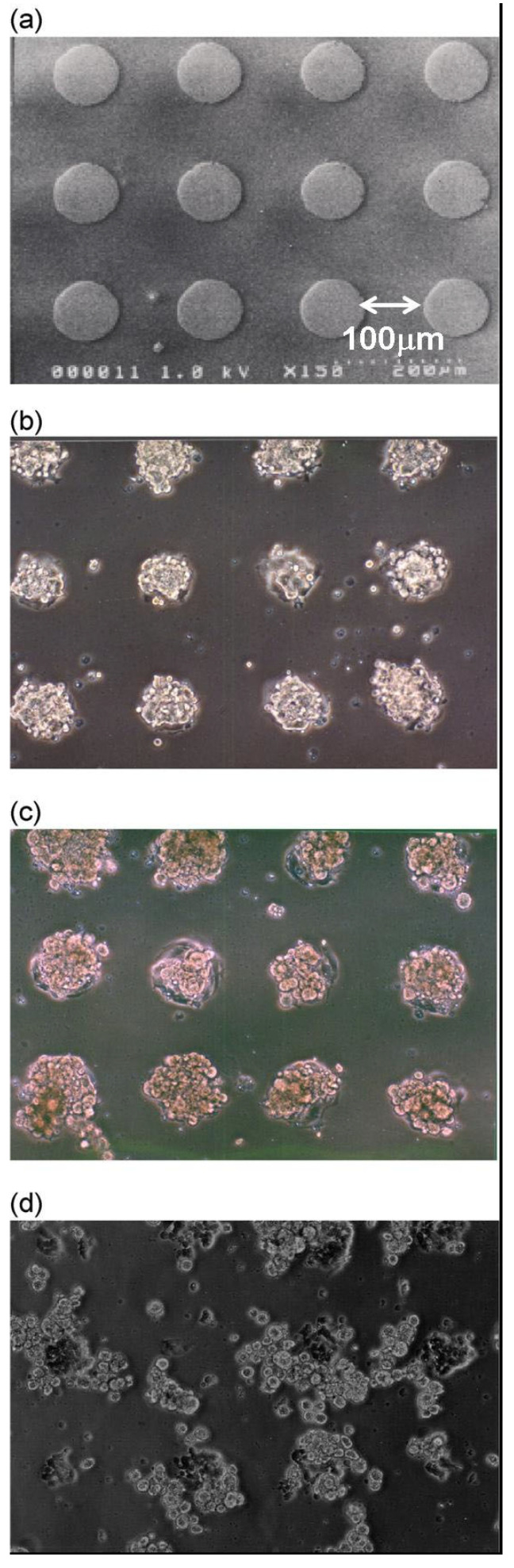
Patterned 3D co-culture of hepatocyte spheroids and endothelial cells (BAECs). (**a**) Micro-patterned α-lactosyl-PEG/PLA-coated dish with *ϕ*100 μm circular domains, spaced in 100-μm intervals. (**b**) Patterned culture of BAECs on the substrate (**a**) for 24 h at 37 °C. (**c**) Organized pattern of hepatocyte spheroids underlaid with BAECs. (**d**) Hepatocytes directly seeded onto substrate (**a**) without pre-adhered BAECs. Reprinted with permission from Ref. [30]. Copyright 2015, John Wiley and Sons.

Spin-coating of polylactic acid (PLA) and α-lactosyl polyethylene glycol (PEG)/PLA block copolymers sequentially onto glass slides, which were hydrophobized by silane coupling treatment and then plasma etching, using a metal mask pattern with circular holes, was performed to fabricate micropatterned PEG substrates with two-dimensional arrays of plasma-etched holes (*ϕ*100-μm) (Figure 3a). Bovine aortic endothelial cells (BAECs) at passage 13 were then cultured on the micro-patterned surfaces at 37 °C for 24 h in a 10% fetal bovine serum medium (Figure 3a). As shown, the BAECs adhered only onto the circular holes, exposing a plasma-etched glass region (Figure 3b). Preferentially adsorbed serum proteins, including cell adhesion proteins such as fibronectin, on the glass regions may promote the glass-affinity of anchorage-dependent BAECs, leading to adhesion. Rat primary hepatocytes in a culture medium were then seeded onto the patterned dishes with cultured endothelial cells. Primary rat hepatocytes generated three-dimensional aggregates of cells within 24 h, only on the circular pattern of previously attached BAECs, forming a two-dimensional array structure of the hepatocyte heterogeneous spheroids (Figure 3c). On the other hand, on the same patterned α-lactosyl-PEG/PLA surface without previously seeded BAECs, hepatocytes adhered to and spread over the entire cell culture substrate, but no spheroids were formed (Figure 3d). These results indicate that BAECs play an important role as a feeder layer for hepatocyte spheroid formation, and that cell activity was well retained in the spheroid by biologically communicating with the underlying BAEC.

**Figure 4:**
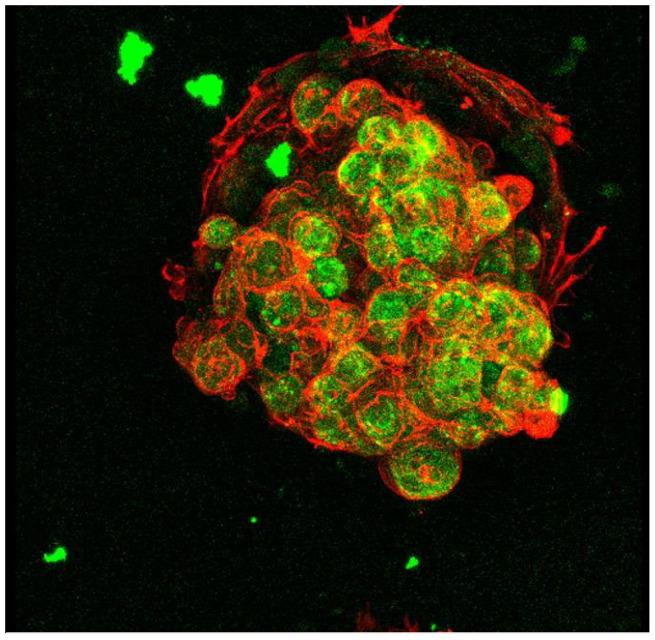
A 3D view of the spheroids shown in Figure 3c, underlaid with endothelial cells as a feeder layer. Reprinted with permission from Ref. [30]. Copyright 2015, John Wiley and Sons.

Heterogeneous hepatocyte spheroids co-cultured with BAECs were evaluated by an immuno-histochemical double staining. In situ fluorescent staining was performed with an anti-rat albumin antibody, to determine characteristic hepatocyte phenotype of albumin synthesis, and a rhodamine-conjugated phalloidin to visualize F-actin. As shown in Figure 4, a multicellular spheroid of hepatocytes in contact with BAECs as a feeder cell is demonstrated by a three-dimensional view. This was reconstructed by overlaying image volumes sliced in two dimensions. Note that the spheroids show strong green fluorescence compared to normal cell monolayers, indicating that they significantly express stable levels of liver-specific function (albumin secretion) even after 3 weeks. It should be noted that these albumin secretions, cytoskeletons, and intercellular junctions remain intact within the spheroid. This is probably due to heterogeneous cell-to-cell interactions through hepatocyte–BAEC contact [60,61,62,63].

To investigate a drug-induced hepatic lesion, in vitro models incorporating primary hepatocytes have been suggested to be more predictable than culture systems relying on liver microsomes. Methods to phenotypically stabilize primary hepatocytes in vitro often depend on the mimesis of the hepatic microenvironment, such as cell-to-cell interactions and cell-to-ECM interactions. In one study, Bhatia et al. [64] incorporated stable hepatocytes into 3D spheroids, which, in turn, could be useful in drug-screening devices such as multi-well plates and a variety of organs-on-a-chip. They first used the micropatterning of rat primary hepatocytes on cell adhesive collagen I, in order to control cell–cell adhesion in two dimensions, then digested the sample by collagenase to produce well-controlled aggregates for 3D encapsulation in the hydrogel, formulated by polyethylene glycol (PEG) diacrylate.

To determine whether the pre-aggregation of hepatocytes into pucks improved liver function after encapsulation in 3D hydrogels, albumin secretion by cells in PEG gels was measured as an indicator of liver phenotype and viability. As a comparison, hepatocytes were randomly seeded on collagen-coated, but unpatterned, six-well plates at a cell density of 600,000 cells/well. This was selected to match the theoretical cell number seeded in wells with a 50 μm island patter, assuming five to six hepatocytes per island. Hepatocytes were randomly seeded in six-well plates (‘‘no pattern’’) or were micropatterned using collagen coated-islands, detached with collagenase, resuspended in hydrogel prepolymer, and photopolymerized into 14 μL disc-shaped samples. Live/dead staining, with calcein AM and ethidium homodimer, of gels encapsulating pucks with spheroid-like cell aggregation structures showed >80% viability after 3 h, indicating that the polymerization process itself was not cytotoxic to the viability (~80%) of the liver cell pucks before post-stripping encapsulation. Secreted albumin in hydrogels containing pucks showed an increasing secretion of albumin for the first week after encapsulation, in comparison with control hydrogels, containing randomly seeded hepatocytes at 8 days. These results indicate that micropatterned hepatocytes forming liver pucks before encapsulation stabilize the phenotype of hepatocytes after encapsulation in a 3D hydrogel.

In addition, in order to help maintain primary hepatocyte function, it has been reported that co-culturing hepatocytes with a second cell type [65,66] and culturing them on surfaces where the adhesion proteins or peptides such as RGDS [67,68] are present is favorable. Therefore, we investigated the incorporation of heterotypic cell–cell interactions and ECM-derived adhesion sites into 3D gels. To achieve co-culture, cell suspensions of J2-3T3 fibroblasts were encapsulated in PEG prepolymer along with hepatocyte pucks. In the resulting gel, J2-3T3 fibroblasts (shown in blue) and hepatocytes were distributed over the entire volume of the gel on a length scale (<100 μm from the nearest hepatocyte), for which previous studies have shown to allow paracrine signaling by soluble factors [69]. Acrylate-functionalized RGDS peptides were covalently attached to the hydrogel network to incorporate cell-adhesive moieties into the gel. A 3D co-culture of fibroblasts and hepatocyte pucks increased albumin secretion more than two-fold when compared to hepatocyte-only controls. It is important to note that the maintenance of albumin secretion over time was prolonged with the cocultivation of fibroblasts. Conversely, adhesion peptides had no significant effect on the albumin generation curve in gels containing RGDS, compared to controls without RGDS, neither in gels containing only hepatocyte pucks nor in hepatocyte pucks containing fibroblasts. These results indicate that supportive signals from J2-3T3 fibroblasts, but not the function of RGDS adhesion peptides, are important for the long-term survival of hepatocytes.

The spheroid formation-based TBB and CBB (tissue- and cell-based biosensors) presented here are expected to be able to respond to environmental disturbances such as toxins and pathogens, in physiologically relevant ways. This miniaturized engineered tissue-like microarray has the ability to rapidly assess the potential health risks of drugs and environmental perturbations, and to predict the effects of exposure. In addition, new techniques for detaching and retrieving arrays of cell-organized structures, such as surface-patterned spheroids, may be particularly important when spheroids are seeded onto 3D scaffolds in order to construct tissue-engineered livers. It is also an effective means of gaining insights into the mechanisms of cell–cell interactions, a central research topic in cell biology. Because cell spheroids offer cell-to-cell interactions and show advantages in terms of their paracrine effects, which can be used to solve clinical and biomedical inquiries, ranging from tissue engineering to disease pathophysiology, they are recently reported to be ideal vehicles for gene delivery. Genetically modified spheroids can enhance specific gene expression to promote tissue regeneration [70,71,72,73,74,75]. Gene deliveries to cell spheroids are achieved via viral vectors or non-viral vectors. It has been shown that genetically modified cell spheroids have the potential to differentiate into bone [76,77,78,79,80,81], cartilage [82,83], vascular tissue, nerve tissue, cardiomyocytes [84,85], skin [86], and skeletal muscle, as well as organs such as the liver [87], in order to replace the diseased organ, as observed in animal and preclinical trials.

### 2.4. Cell Aggregates for Tissue Engineering

Artificial microtissues can also be fabricated by inducing the aggregation of one or more cell type [88]. These microtissues and arrays may be useful for studies such as tissue engineering of the pancreatic, liver, vascular, and cardiac tissues, as well as comprehensive drug screening. Cell stacking has been used for cardiac tissue engineering by assembling multiple sheets of cardiomyocytes [89], as well as vascular engineering by fabricating cylindrically rolled sheets of endothelial cells [90]. Such an approach may satisfy certain tissue engineering applications, but lacks the tissue formation that can reproduce more complicated organ structures. Microscale efforts may provide a novel culture system for this challenge, by serving as templates for the reproducible creation of complex microtissues. For example, hepatocyte spheroids have been formed by combining microcontact printing and micromachining [91]. Recently, microwells made of nonadherent PEG have been used as templates for the formation of various types of cell assemblies, including ES cells [92]. This approach aims to overcome the drawbacks faced by hanging drop and suspension culture methods [88], by controlling the size, shape, and other characteristics of cell assemblies in a scalable manner. Th controlled aggregation of embryoid bodies can induce the differentiation of ES cells, which may also be used as a novel technique in order to generate more homogeneous cultures. Furthermore, the template-based assembly of cells may allow for the organization of multiple cell types into specific shapes relative to each other in these cohesive tissue sections. Combining such methodologies with biomaterials such as crosslinked polymer hydrogels and μ-TAS may allow for the creation of more complex therapeutic tissue organizations.

In tissue engineering applications, after the spheroid cells are retrieved from the culture substrate, they are subsequently incorporated into three-dimensional polymer scaffolds that act similarly to the natural extracellular matrices that make up tissues. These scaffolds safely deliver cells to the site of injury in the patient’s body, provide a space for new tissue formation, and have the potential to control the formation and function of engineered tissues [93,94]. In light of previous tissue engineering approaches, an important element is the polymeric scaffold, which serves as an extracellular matrix. This artificial extracellular matrix is composed of various amino acid- and sugar-based macromolecules that organize cells, control tissue structure, regulate cellular function, and allow the diffusion of nutrients, metabolites, and growth factors that control cell-to-cell communication [95]. Various types of polymers have been three-dimensionally formulated to be utilized as suitable cell scaffolds [96]. Among them, hydrogels have found numerous applications in tissue engineering, because they have structural similarity to the macromolecular-based components in the body and are considered biocompatible [97]. Hydrogels, whose development is currently expected in the field of tissue engineering, are classified into two categories, depending on whether they are naturally or synthetically derived. Hydrogels derived from natural polymers tend to have strong interactions with cells, along with suitable cell proliferation and differentiation. However, hydrogels from natural polymers have limitations in supporting the reproduction of tissue function, and approaches have been taken to modify natural polymers or supplement their structure with various synthetic polymers. Various synthetic polymers have molecular properties that allow for the fine-tuning of their chemical and physical properties, for their use in such applications.

Here, we will discuss typical natural polymers, divided into polysaccharides and polypeptides, and synthetic polymers, which have been widely used for hydrogel formation, as well as their properties and applications.

## 3. Hydrogels as Three-Dimensional Cell Scaffolds

### 3.1. Synthetic Polymers

The specific applications of polymer scaffold materials to targeted tissue regeneration require certain modifications to the polymer structure. This reorganization is quite difficult with natural polymers, and hence, synthetic polymers, such as aliphatic polyesters, including poly(glycolic acid) (PGA), poly(lactic acid) (PLA), their copolymers (PLGA), and ε-polycaprolactone (PCL), are widely used in tissue engineering. PCL is a relatively low-cost, resilient, biodegradable synthetic polymer, and is approved by the USA Food and Drug Administration (FDA). PLGA is another FDA-approved synthetic biopolymer widely applied in tissue regeneration. The distinctive advantages of PLGA are its biocompatibility and degradability, which can be fine-tuned to express the desired regenerative tissue properties.

### 3.2. Biopolymers

In tissue engineering, biocompatible and biodegradable natural materials of biological origin are used as cell scaffolds in order to provide the appropriate bioactive environment and necessary mechanical support to the diseased site, to promote new tissue growth. Because of its excellent biocompatibility and low immunological infectious effects, natural biopolymers, including polysaccharides and polypeptides such as collagen, chitosan, alginate, silk, fucoidan, elastin, gelatin, hyaluronic acid, are extensively used in regenerative medicine.

#### 3.2.1. Polysaccharides

**Figure 5:**
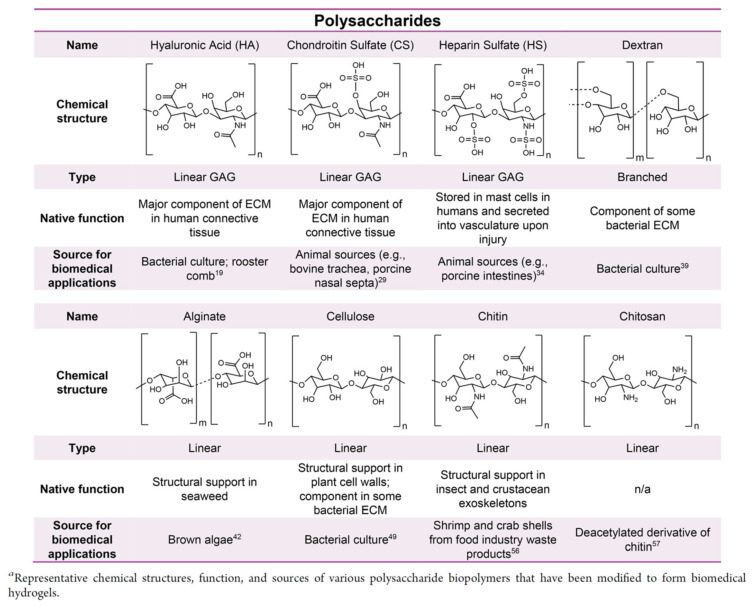
Polysaccharide-Based Biopolymers ^a^. Reprinted with permission from Ref. [98]. Copyright 2015, American Chemical Society.

Polysaccharides are substances composed of a number of monosaccharide molecules joined by glycosidic bonds, and are composed of repeating units of monosaccharides or disaccharides, providing important structural and biological functionality in living organisms (Figure 5). Polysaccharides are obtained as biosynthetic products by living organisms, and are often isolated from renewable resources, such as plants and microorganisms. Hydrogels with a wide range of functions have been developed by the cross-linking of chemically modified polysaccharides.

#### 3.2.2. Polypeptide

**Figure 6:**
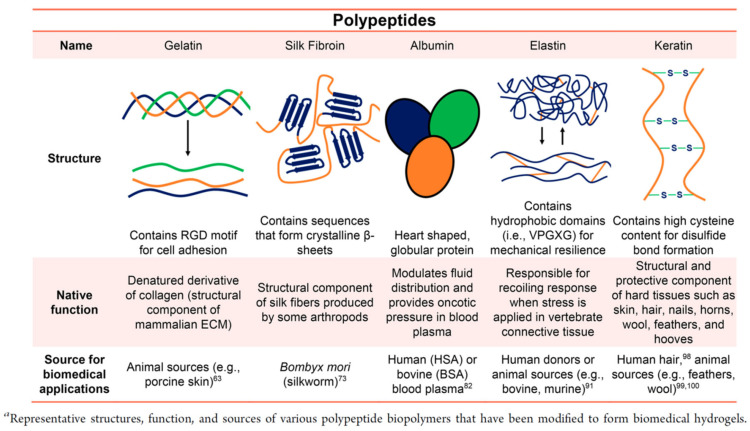
Polypeptide-Based Biopolymers ^a^. Reprinted with permission from Ref. [98], Copyright 2015, American Chemical Society.

A polypeptide is a biopolymer consisting of linear repeating chains of amino acids; if it consists of more than 50 amino acids, it is defined as a protein (Figure 6). Hydrogels made from polypeptides have many cell interaction sites and can mimic the function of the extracellular matrix, making them suitable for biomedical applications. Polypeptides can be isolated from animal or plant sources or synthetically biomanufactured using peptide synthesizers, or recombinant protein production, as a function of total host cell biomass synthetic capacity.

### 3.3. Methodologies of Cross-Linking

Gelation is caused by crosslinking of polymers to form a polymer network with an internal solvent. There are two main types of crosslinking: chemical crosslinking and physical crosslinking. Chemical crosslinking is formed by covalent bonding of functional groups within the biopolymer or synthetic polymer, and is generally strong and stable. In contrast, physical cross-linking is formed by non-covalent bonding, such as hydrogen bonding, between polar polymer chains. Although the mechanical strength may be low, gels with self-healing and shear-thinning properties are often obtained due to the reversibility of the physical interaction. These typical methods are described below.

#### 3.3.1. Chemical Cross-Linking

In general, covalent bonds are stable and enable the synthesis of hydrogels with high mechanical strengths. Among covalent bonds, the introduction of dynamic covalent bonds, which are reversible reactions, makes it possible to give gels self-healing properties, while maintaining their high mechanical strength. In addition, the gelation and degradation behavior may be controlled in a stimulus-responsive manner. Because of these advantages, dynamic covalent gels have been considered for many applications, such as scaffold materials in tissue engineering and drug-delivery systems. In this section, we will discuss the covalent click chemistry, active-ester reaction and dynamic covalent Schiff bases, as representative examples.

##### Click Chemistry

Click chemistry was proposed by K.B. Sharpless as a versatile, substrate-specific reaction to proceed at a high yield and without by-products. The advantages of click chemistry are that it is simple to operate, and the substrates and products are stable in water and oxygen and proceed under a wide variety of reaction conditions. Because of its biocompatibility, reliability, and bio-orthogonal specificity, it is widely used in biomedical applications, such as biomaterial engineering and drug discovery. Typical click chemistry methods used for hydrogel cross-linking include the thiol-ene Michael addition reaction, thiol-ene radical addition reaction, azide–alkyne reaction, and tetrazine–norbornene cycloaddition.

##### Active-Ester Reaction

When forming hydrogels, it is common to use active esters of carboxylic acids for milder cross-linking that does not lead to cytotoxicity. After converting the carboxylic acids in the polymer to N-hydroxy succinimide (NHS) esters, these esters are readily attacked when mixed with free amine groups, which are then converted into stable amide bonds. The disadvantage of this cross-linking is that the NHS-functionalized polymer must be thoroughly mixed with the amino-group-containing polymer immediately prior to use when used in water, because of the rapid hydrolysis rate of the NHS functional group.

##### Schiff Bases

A Schiff base is a bond formed by the condensation reaction of a carbonyl group with a primary amine, and is represented by the general formula R_1_R_2_C = NR_3_. Only water is produced as a byproduct. The reversible reaction of Schiff base formation proceeds under mild conditions and is pH-dependent. In the biomaterials field, hydrogels are formed by mixing aldehyde-functionalized and amine-functionalized polymers. It is also known that the neighboring atoms of primary amines used for Schiff base formation affect the stability of cross-linking. A Schiff base with a nitrogen atom next to the primary amine is called a hydrazone bond, which can form a more stable hydrogel than a normal Schiff base [99].

#### 3.3.2. Physical Cross-Linking

Physical cross-links are formed by physical interactions between biomacromolecules, such as hydrogen bonding, hydrophobic interactions, and electrostatic attraction. Although mechanical strength may be lower than for gels cross-linked by chemical cross-linking, physical cross-linking can be used to enhance the cell migration and diffusion of cells [99]. In addition, physical interactions are reversible and can impart self-healing properties to hydrogels. Physical cross-linking is usually formed by the one-pot mixing of two or more components, without the need for an external catalyst or initiator.

#### 3.3.3. Injectable IPN Gels as New and Minimally Invasive Applications

**Figure 7:**
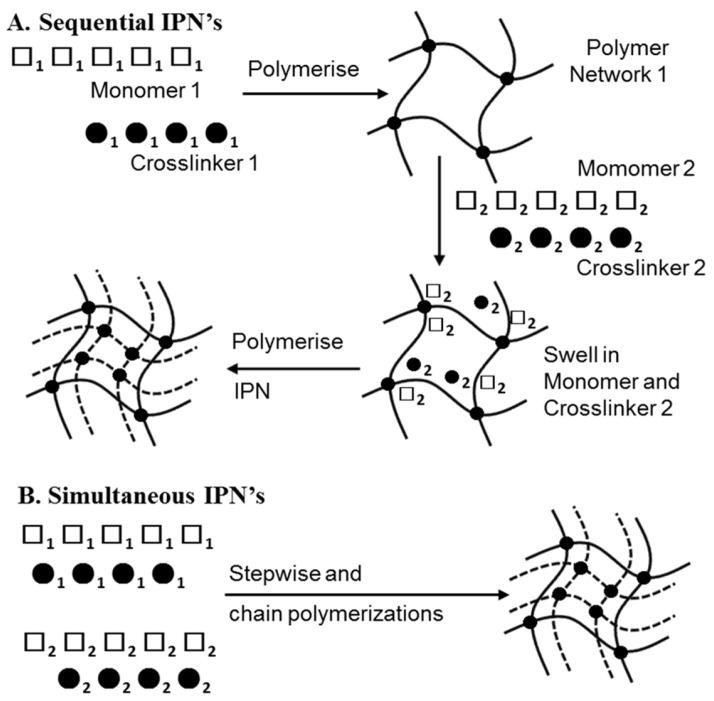
Two popular methods of preparing interpenetrating polymer networks. Reprinted with permission from Ref. [101]. Copyright 2015, Elsevier.

**Figure 8:**
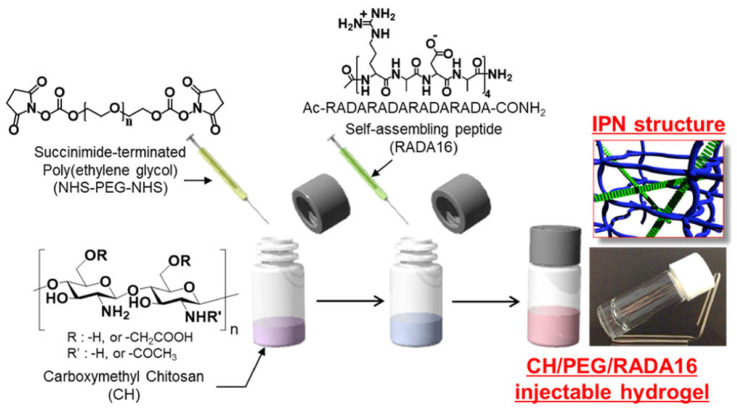
Schematic illustration of the “One-Pot” synthesis of the CH/PEG/RADA16 injectable hydrogel, with the IPN structure. Reprinted with permission from Ref. [104] Copyright 2015, American Chemical Society.

Interpenetrating polymer network (IPN) gels have attracted much attention in recent years due to their high utility for many biomedical applications. An IPN is defined by the IUPAC as a “Polymer comprising two or more polymer networks which are at least partially interlaced on a molecular scale, but not covalently bonded to each other and cannot be separated unless chemical bonds are broken” [100]. A simple mixture of two or more different crosslinked polymers is not an interpenetrating polymer network. An IPN is further defined by the reaction mechanism by which it is polymerized. When an IPN is prepared by a mechanism, in which the second component network is synthesized after the first component network is completely polymerized, the IPN is sometimes referred to as a sequential IPN. If an IPN is prepared by a process in which both component networks are polymerized simultaneously, the IPN is referred to as a simultaneous IPN. As shown in Figure 7, the synthesis types of IPN are as follows: (A) sequential IPN, usually done by swelling the single first network in a solution containing a mixture of monomers, initiators and activators, with or without a cross-linker, followed by the formation of a second network; and (B) simultaneous IPN, where both network precursors are mixed, and the two networks are synthesized simultaneously by independent, and non-interfering routes, such as chain and stepwise polymerization. [101,102,103]. The reason for the development of these structures is to combine the favorable properties of each constituent polymer within a new overall hydrogel network, and to modulate the mechanical and biological properties of each constituent. Generally, the resulting hydrogel system possesses enhanced properties, due to synergistic effects. In addition, by using multiple materials and designing gels that take advantage of their characteristics, gels with numerous functions have been developed, such as those with high mechanical strength, affinity to cells, self-healing properties, and chondrogenic differentiation properties. In sequential IPN, gels are usually synthesized through multiple steps: first, polymer network I is synthesized. Then, monomer II, plus the crosslinker and activator, are swollen into network I and polymerized in situ with external stimuli to form the second network. However, those activators and stimuli are likely to be toxic in in situ gels that are injected into the body. Therefore, a new strategy of using injectable hydrogels was devised, in order to synthesize IPN gels in a one-pot, injectable, and time-dependent manner by utilizing two different network-forming driving forces [104]. In the tissue engineering field, an innovative alternative approach to cell delivery is the use of polymers (i.e., hydrogels) that are fluid, to the extent that they can be injected into the body. This approach allows the clinician to transplant the cell and polymer combinations in a minimally invasive manner. Among hydrogels, injectable gels, which form in situ when administered in vivo, are attracting attention as carriers for drug delivery and as scaffolds for tissue engineering. Gels containing cells, therapeutic drugs, growth factors, and other substances can be easily prepared by simply mixing them with a gel precursor polymer solution and injecting them. Ishikawa et al. moderately chemically cross-linked the amino groups of chitosan (CH) with succinimidated poly(ethylene glycol) (NHS-PEG-NHS) gels, followed by addition of the self-aggregating peptide chain RADA16 to form IPNs, which showed high utility and safety in terms of mechanical strength and chondrogenic differentiation properties (Figure 8). This structure is formed by the physical cross-linking of the self-assembling peptide chain RADA16, immediately after the three-component mixture of RADA16, CH, and NHS-PEG-NHS, followed by the chemical cross-linking of chitosan and polyethylene glycol. This one-pot and in situ method of synthesizing IPN gels allows for a wide variety of material combinations, and is expected to be a widely applicable technology, not only in tissue regeneration, but also in drug delivery, diagnosis, etc.

This review highlights the current state of spheroids for clinical use and demonstrates their immense capacity for tissue regeneration. However, the clinical translation of spheroids has lagged behind promising preclinical outcomes, due to hurdles in their formation, instruction, and use that have yet to be overcome. This review will contribute to future clinical medicine in terms of the successful development of methods to accelerate spheroid formation, reliable in situ instructions employing hydrogels as cell scaffolds, and the characterization, control, and utilization of their secreted factors. By establishing these techniques, spheroids may become an advanced cell therapy for regenerative medicine.

## 4. Conclusions

Many of the techniques used for cell patterning fall into two categories; passive patterning, by random seeding on surfaces modified with high-cell-affinity or cell-exclusive regions, and active deposition, or direct printing, of cells onto surfaces via light or electrical forces. Although only the former category is considered in this paper, we believe that there is a great future potential for combining certain techniques with the goal of solving many of the problems inherent in the long-term culture of active cell arrays.

Microfabrication by dry etching and photolithography is expanding rapidly, and “soft lithography” is finding applications in the biotechnology fields [105]. A number of microfluidic devices have also been developed and are rapidly finding applications in the biomedical and pharmaceutical industries [106]. In particular, the formation of multicellular aggregates as spheroids was suggested to be important in long-term drug screening and tissue engineering. Cirrhosis is an irreversible liver injury, in which healthy tissue is replaced by scar tissue and liver function is impaired. There is no cure, and new treatments are urgently needed, as current therapies only prevent further liver damage. We propose a new and novel approach in which autologous 3D spheroids composed of mesenchymal stem cells (MSCs) or human hepatocytes are injected into the body via an injectable hydrogel. This approach allows clinicians to transplant cell and polymer combinations in a minimally invasive manner. When compared to the surgeon, who makes incisions (cuts) sufficiently large to enable placement of the polymer/cell constructs, MSC spheroids, which are delivered to the body in an injectable hydrogel, offer significant advantages in tissue–structure formation and paracrine factor secretion.

## References

[1] Sivashanmugam A., Kumar R.A., Priya M.V., Nair S.V., Jayakumar R. (2015). An overview of injectable polymeric hydrogels for tissue engineering. Eur. Polym. J..

[2] Jeon N.L., Baskaran H., Detringer S.K.W., Whitesides G.M., Water L.V., Toner M. (2002). Neutrophil chemotaxis in linear and complex gradients of interleukin-8 formed in a microfabricated device. Nat. Biotechnol..

[3] Britland S., Clark P., Connolly P., Moores G. (1992). Micropatterned substratum adhesiveness: A model for morphogenetic cues controlling cell behavior. Ex. Cell Res..

[4] Healy K.E., Lom B., Hockberger P.E. (1994). Spatial distribution of mammalian cells dictated by material surface chemistry. Biotechnol. Bioeng..

[5] Hickman J.J., Bhatia S.K., Quong J.N., Shoen P., Stenger D.A., Pike C.J., Cotman C.W. (1994). Rational pattern design for in-vitro cellular networks using surface photochemistry. J. Vac. Sci. Technol. A.

[6] Bekos E.J., Ranien J.P., Aebischer P., Gardella J.A., Bnght F.V. (1995). Structural Changes of Bovine Serum Albumin upon Adsorption to Modified Fluoropolymer Substrates Used for Neural Cell Attachment Studies. Langmuir.

[7] Singhvi R., Kumar A., Lopez G.P., Stehanopoulos G.N., Wang D.I.C., Whitesides G.M., Ingber D.E. (1994). Engineering Cell Shape and Function. Science.

[8] Spargo B.J., Testoff M.A., Nielsen T.B., Stenger D.A., Hickman J.J., Rudolph A.A. (1994). Spatially controlled adhesion, spreading, and differentiation of endothelial cells on self-assembled molecular monolayers. Proc. Natl. Acad. Sci. USA.

[9] Stenger D.A., Georger J.H., Dulcey C.S., Hickman J.J., Rudolph A.S., Nielsen T.B., McCort S.M., Calvert J.M. (1992). Coplanar molecular assemblies of amino- and perfluorinated alkylsilanes: Characterization and geometric definition of mammalian cell adhesion and growth. J. Am. Chem. Soc..

[10] van den Berg A., Lammerink T.S.J., Manz A., Becker H. (1998). Micro Total Analysis Systems: Microfluidic Aspects, Integration Concept and Applications. Microsystem Technology in Chemistry and Life Science. Topics in Current Chemistry.

[11] Manz A., Graber N., Widmer H.M. (1990). Miniaturized total chemical analysis systems: A novel concept for chemical sensing. Sens. Actuat. B Chem..

[12] Dittrich P.S., Manz A. (2006). Lab-on-a-chip: Microfluidics in drug discovery. Nat. Rev. Drug Discov..

[13] Bhatia S.N., Balis U.J., Yarmush M.L., Toner M. (1999). Effect of cell-cell interactions in preservation of cellular phenotype: Cocultivation of hepatocytes and nonparenchymal cells. FASEB J..

[14] Folch A., Toner M. (2000). Microengineering of cellular interactions. Annu. Rev. Biomed. Eng..

[15] Tsang V.L., Bhatia S.N. (2004). Three-dimensional tissue engineering. Adv. Drug. Deliv. Rev..

[16] Wang W., Itaka K., Ohba S., Nishiyam N., Chung U., Yamasaki Y., Kataoka K. (2009). 3D spheroid culture system on micropatterned substrates for improved differentiation efficiency of multipotent mesenchymal stem cells. Biomaterials.

[17] Chen C.S., Mrksich M., Huang S., Whitesides G.M., Ingber D.E. (1997). Geometric Control of Cell Life and Death. Science.

[18] Zamir E., Katz B.Z., Aota K.M., Yamada K.M., Geiger B., Kam Z. (1999). Molecular diversity of cell-matrix adhesions. J. Cell Sci..

[19] Geiger B., Bershadsky R., Pankov R., Yamada K.M. (2001). Transmembrane crosstalk between t he extracellular matrix and the cytoskeleton. Nat. Rev. Mol. Cell Biol..

[20] Chou L., Firth J.D., Uitto V.J., Brunette D.M. (1995). Substratum surface topography alters cell shape and regulates fibronectin mRNA level, mRNA stability, secretion and assembly in human fibroblasts. J. Cell. Sci..

[21] Clark P., Connolly P., Curtis A.S.G., Dow J.A.T., Wilkinson C.D.W. (1990). Topographical control of cell behaviour: II. Multiple grooved substrata. Development.

[22] Stenger D.A., Gross G.W., Keefer E.W., Shaffer K.M., Andreadis J.D., Ma W., Pancrazio J.J. (2001). Detection of physiologically active compounds using cell-based biosensors. Trends Biotechnol..

[23] Kononen J., Bubendorf L., Kallioniemi A., Barlund M., Schraml P., Leighton S., Torhorst J., Mihatsch M.J., Sauter G., Kallioniemi O.P. (1998). Tissue microarrays for high-throughput molecular profiling of tumor specimens. Nat. Med..

[24] Ziauddin J., Sabatini D.M. (2001). Microarrays of cells expressing defined cDNAs. Nature.

[25] Michalopoulos G.K., DeFrances M.C. (1997). Liver regeneration. Science.

[26] Anderson D.G., Levenberg S., Langer R. (2004). Nanoliter-scale synthesis of arrayed biomaterials and application to human embryonic stem cells. Nat. Biotechnol..

[27] Revzin A., Tompkins R.G., Toner M. (2003). Surface Engineering with Poly(ethylene glycol) Photolithography to Create High-Density Cell Arrays on Glass. Langmuir.

[28] Thielecke H., Mack A., Robitzki A. (2001). A multicellular spheroid-based sensor for anti-cancer therapeutics. Biosen. Bioelect..

[29] Mack A.R., Thielecke H., Robitzki A.A. (2002). 3D-biohybrid systems: Applications in drug screening. Trends Biotechnol..

[30] Otsuka H., Hirano A., Nagasaki Y., Okano T., Horiike Y., Kataoka K. (2004). Two-dimensional multiarray formation of hepatoytes sphroids on a microfabricated PEG-brush surface. ChemBioChem.

[31] Koudan E.V., Bulanova E.A., Pereira F.D.A.S., Parfenov V.A., Kasyanov V.A., Hesuani Y.D., Mironov V.A. (2016). Patterning of tissue spheroids biofabricated from human fibroblasts on the surface of electrospun polyurethane matrix using 3D bioprinter. Int. J. Bioprinting.

[32] Ko J., Ahn J., Kim S., Lee Y., Lee J., Park D., Jeon N.L. (2019). Tumor spheroid-on-a-chip: A standardized microfluidic culture platform for investigating tumor angiogenesis. Lab Chip.

[33] Yeh H.Y., Hsieh F.Y., Hsu S.H. (2016). Self-patterning of adipose-derived mesenchymal stem cells and chondrocytes cocultured on hyaluronan-grafted chitosan surface. Biointerphases.

[34] Berg I.C., Mohagheghian E., Habing K., Wang N., Underhill G.H. (2021). Microtissue Geometry and Cell-Generated Forces Drive Patterning of Liver Progenitor Cell Differentiation in 3D. Adv. Healthcare Mater..

[35] Su C., Chuah Y.J., Ong H.B., Tay H.M., Dalan R., Hou H.W. (2021). A facile and scalable hydrogel patterning method for microfluidic 3D cell culture and spheroid-in-gel culture array. Biosensors.

[36] Heo D.N., Hospodiuk M., Ozbolat I.T. (2019). Synergistic interplay between human MSCs and HUVECs in 3D spheroids laden in collagen/fibrin hydrogels for bone tissue engineering. Acta Biomater..

[37] Ho S.S., Keown A.T., Addison B., Leach J.K. (2017). Cell Migration and Bone Formation from Mesenchymal Stem Cell Spheroids in Alginate Hydrogels Are Regulated by Adhesive Ligand Density. Biomacromolecules.

[38] Yang M., He S., Su Z., Yang Z., Liang X., Wu Y. (2020). Thermosensitive Injectable Chitosan/Collagen/β-Glycerophosphate Composite Hydrogels for Enhancing Wound Healing by Encapsulating Mesenchymal Stem Cell Spheroids. ACS Omega.

[39] Kim S., Kim E.M., Yamamoto M., Park H., Shin H. (2020). Engineering Multi-Cellular Spheroids for Tissue Engineering and Regenerative Medicine. Adv. Healthc. Mater..

[40] Baptista L.S., Kronemberger G.S., Côrtes I., Charelli L.E., Matsui R.A.M., Palhares T.N., Sohier J., Rossi A.M., Granjeiro J.M. (2018). Adult Stem Cells Spheroids to Optimize Cell Colonization in Scaffolds for Cartilage and Bone Tissue Engineering. Int. J. Mol. Sci..

[41] Tseng T.C., Wong C.W., Hsieh F.Y., Hsu S.H. (2017). Biomaterial Substrate-Mediated Multicellular Spheroid Formation and Their Applications in Tissue Engineering. Biotechnol. J..

[42] Ho S.S., Murphy K.C., Binder B.Y.K., Vissers C.B., Leach J.K. (2016). Increased Survival and Function of Mesenchymal Stem Cell Spheroids Entrapped in Instructive Alginate Hydrogels. Stem Cells Transl. Med..

[43] Nilforoushzadeh M.A., Yazdi M.K., Ghavami S.B., Farokhimanesh S., Amirabad L.M., Zarrintaj P., Saeb M., Hamblin M.R., Zare M., Mozafari M. (2020). Mesenchymal Stem Cell Spheroids Embedded in an Injectable Thermosensitive Hydrogel: An In Situ Drug Formation Platform for Accelerated Wound Healing. ACS Biomater. Sci. Eng..

[44] Gionet-Gonzales M.A., Leach J.K. (2018). Engineering principles for guiding spheroid function in the regeneration of bone, cartilage, and skin. Biomed. Mater..

[45] Griffin K.H., Fok S.W., Leach J.K. (2022). Strategies to capitalize on cell spheroid therapeutic potential for tissue repair and disease modeling. NPJ Regen. Med..

[46] Hoch A.I., Mittal V., Mitra D., Vollmer N., Zikry C.A., Leach J.K. (2016). Cell-secreted matrices perpetuate the bone-forming phenotype of differentiated mesenchymal stem cells. Biomaterials.

[47] Laschke M.W., Menger M.D. (2017). Life is 3D: Boosting Spheroid Function for Tissue Engineering. Trends Biotechnol..

[48] Ong C.S., Zhou X., Han J., Huang C.Y., Nashed A., Khatri S., Mattson G., Fukunishi T., Zhang H., Hibino N. (2018). In vivo therapeutic applications of cell spheroids. Biotechnol. Adv..

[49] Shen J.X., Youhanna S., Shafagh R.Z., Kele J., Lauschke V.M. (2020). Organotypic and Microphysiological Models of Liver, Gut, and Kidney for Studies of Drug Metabolism, Pharmacokinetics, and Toxicity. Chem. Res. Toxicol..

[50] Rodrigues T., Kundu B., Correia J.S., Kundu S.C., Oliveira J.M., Reis R.L., Correlo V.M. (2018). Emerging tumor spheroids technologies for 3D in vitro cancer modeling. Pharmacol. Ther..

[51] Park J., Choe G., Oh S., Lee J.Y. (2020). In Situ Formation of Proangiogenic Mesenchymal Stem Cell Spheroids in Hyaluronic Acid/Alginate Core–Shell Microcapsules. ACS Biomater. Sci. Eng..

[52] Whitehead J., Griffin K.H., Gionet-Gonzales M., Vorwald C.E., Cinque S.E., Leach J.K. (2021). Hydrogel mechanics are a key driver of bone formation by mesenchymal stromal cell spheroids. Biomaterials.

[53] Ahmad T., Byun H., Lee J., Kumar S., Perikamana M., Shin Y.M., Kim E.M., Shin H. (2020). Stem cell spheroids incorporating fibers coated with adenosine and polydopamine as a modular building blocks for bone tissue engineering. Biomaterials.

[54] Lee W., Choi J.H., Lee S., Song J.E., Khang G. (2020). Fabrication and characterization of silk fibroin microfiberincorporated bone marrow stem cell spheroids to promote cell-cell interaction and osteogenesis. ACS Omega.

[55] Ahmad T., Shin H.J., Lee J., Shin Y.M., Perikamana S.K.M., Park S.Y., Jung H.S., Shin H. (2018). Fabrication of in vitro 3D mineralized tissue by fusion of composite spheroids incorporating biomineral-coated nanofibers and human adipose-derived stem cells. Acta Biomater..

[56] Lee J., Lee S., Ahmad T., Perikamana S.K.M., Lee J., Kim E.M., Shin H. (2020). Human adiposederived stem cell spheroids incorporating platelet-derived growth factor (PDGF) and bio-minerals for vascularized bone tissue engineering. Biomaterials.

[57] Lee N.-H., Bayaraa O., Zechu Z., Kim H.S. (2021). Biomaterials-assisted spheroid engineering for regenerative therapy. BMB Rep..

[58] Hong Y., Chen J., Fang H., Li G., Yan S., Zhang K., Wang C., Yin J. (2020). All-in-one hydrogel realizing adipose-derived stem cell spheroid production and in vivo injection via “Gel-Sol” transition for angiogenesis in hind limb ischemia. ACS Appl. Mater. Interfaces.

[59] Ran P., Chen W., Wei J., Qiu B., Chen M., Xie S., Li X. (2020). Macrophage spheroids with chronological phenotype shifting to promote therapeutic angiogenesis in critical limb ischemia. ACS Appl. Bio Mater..

[60] Matsumoto K., Yoshitomi H., Rossant J., Zaret K.S. (2001). Liver organogenesis promoted by endothelial cells prior to vascular function. Science.

[61] Zaret K.S. (2002). Regulatory phases of early liver development: Paradigms of organogenesis. Nat. Rev. Genet..

[62] LeCouter J., Moritz D.R., Li B., Phillips G.L., Liang H., Gerber H.P., Hillan K.J., Ferrara N. (2003). Angiogenesis-independent endothelial protection of liver: Role of VEGFR-1. Science.

[63] Ferrara N., Gerber H.P., LeCouter J. (2003). The biology of VEGF and its receptors. Nat. Med..

[64] Cheri Y.L., Kelly R.S., Robert E.S., Brian S.A., Joanne H.H., Sangeeta N.B. (2014). Micropatterned Cell–Cell Interactions Enable Functional Encapsulation of Primary Hepatocytes in Hydrogel Microtissues. Tissue Eng. Part A.

[65] Khetani S.R., Bhatia S.N. (2008). Microscale culture of human liver cells for drug development. Nat. Biotechnol..

[66] Lu H.F., Chua K.N., Zhang P.C., Lim W.S., Ramakrishna S., Leong K.W., Mao H.Q. (2005). Three-dimensional co-culture of rat hepatocyte spheroids and NIH/3T3 fibroblasts enhances hepatocyte functional maintenance. Acta. Biomater..

[67] Underhill G.H., Chen A.A., Albrecht D.R., Bhatia S.N. (2007). Assessment of hepatocellular function within PEG hydrogels. Biomaterials.

[68] Chen A.A., Thomas D.K., Ong L.L., Schwartz R.E., Golub T.R., Bhatia S.N. (2021). Humanized mice with ectopic artificial liver tissues. Proc. Natl. Acad. Sci. USA.

[69] Hui E.E., Bhatia S.N. (2007). Micromechanical control of cell-cell interactions. Proc. Natl. Acad. Sci. USA.

[70] Miyoshi H., Stappenbeck T.S. (2013). In vitro expansion and genetic modification of gastrointestinal stem cells in spheroid culture. Nat. Protoc..

[71] Kim H.J., Alam Z., Hwang J.W., Hwang Y.H., Kim M.J., Yoon S., Byun Y., Lee D.Y. (2013). Optimal formation of genetically modified and functional pancreatic islet spheroids by using hanging-drop strategy. Transplant. Proc..

[72] Kim H., Park H.J., Choi H., Chang Y., Park H., Shin J., Kim J., Lengner C.J., Lee Y.K., Kim J. (2019). Modeling G2019S-LRRK2 sporadic parkinson’s disease in 3D midbrain organoids. Stem Cell Rep..

[73] Schwank G., Koo B.K., Sasselli V., Dekkers J.F., Heo I., Demircan T., Sasaki N., Boymans S., Cuppen E., van der Ent C.K. (2013). Functional repair of CFTR by CRISPR/cas9 in intestinal stem cell organoids of cystic fibrosis patients. Cell Stem Cell.

[74] Geurts M.H., de Poel E., Amatngalim G.D., Oka R., Meijers F.M., Kruisselbrink E., van Mourik P., Berkers G., De Winter-de Groot K.M., Michel S. (2020). CRISPR-based adenine editors correct nonsense mutations in a cystic fibrosis organoid biobank. Cell Stem Cell.

[75] Han S., Lee C., Im J., Kim J., Kim J., Kim S., Cho Y., Kim E., Kim Y., Ryu J. (2021). Targeted suicide gene therapy for liver cancer based on ribozyme-mediated RNA replacement through post-transcriptional regulation. Mol. Ther. Nucl. Acid..

[76] Malek-Khatabi A., Javar H., Dashtimoghadam E., Ansari S., Hasani-Sadrabadi M., Moshaverinia A. (2020). In situ bone tissue engineering using gene delivery nanocomplexes. Acta Biomater..

[77] Liang Z., Luo Y., Lv Y. (2020). Mesenchymal stem cell-derived microvesicles mediate BMP2 gene delivery and enhance bone regeneration. J. Mater. Chem. B.

[78] Loozen L., Kruyt M., Kragten A., Schoenfeldt T., Croes M., Oner C., Dhert W., Alblas J. (2019). BMP-2 gene delivery in cell-loaded and cell-free constructs for bone regeneration. PLoS ONE.

[79] Curtin C., Tierney E., McSorley K., Cryan S., Duffy G., O’Brien F. (2015). Combinatorial gene therapy accelerates bone regeneration: Non-viral dual delivery of VEGF and BMP2 in a collagen-nanohydroxyapatite scaffold. Adv. Health. Mater..

[80] Celik N., Kim M.H., Hayes D.J., Ozbolat I.T. (2021). miRNA induced co-differentiation and cross-talk of adipose tissue-derived progenitor cells for 3D heterotypic prevascularized bone formation. Biofabrication.

[81] Celik N., Kim M.H., Yeo M., Kamal F., Hayes D.J., Ozbolat I.T. (2022). miRNA induced 3D bioprinted-heterotypic osteochondral interface. Biofabrication.

[82] Chen J., Chen H., Li P., Diao H., Zhu S., Dong L., Wang R., Guo T., Zhao J., Zhang J. (2011). Simultaneous regeneration of articular cartilage and subchondral bone in vivo using MSCs induced by a spatially controlled gene delivery system in bilayered integrated scaffolds. Biomaterials.

[83] Yang X., Shang H., Katz A., Li X. (2013). A modified aggregate culture for chondrogenesis of human adipose-derived stem cells genetically modified with growth and differentiation factor 5. Biores. Open Access.

[84] Moon H., Joo M., Mok H., Lee M., Hwang K., Kim S., Jeong J., Choi D., Kim S. (2014). MSC-based VEGF gene therapy in rat myocardial infarction model using facial amphipathic bile acid-conjugated polyethyleneimine. Biomaterials.

[85] Chen Y., Zhao Y., Chen W., Xie L., Zhao Z., Yang J., Chen Y., Lei W., Shen Z. (2017). MicroRNA-133 overexpression promotes the therapeutic efficacy of mesenchymal stem cells on acute myocardial infarction. Stem Cell Res. Ther..

[86] Ebner-Peking P., Krisch L., Wolf M., Hochmann S., Hoog A., V’ari B., Muigg K., Poupardin R., Scharler C., Schmidhuber S. (2021). Self-assembly of differentiated progenitor cells facilitates spheroid human skin organoid formation and planar skin regeneration. Theranostics.

[87] Guye P., Ebrahimkhani M.R., Kipniss N., Velazquez J.J., Schoenfeld E., Kiani S., Griffith L.G., Weiss R. (2016). Genetically engineering self-organization of human pluripotent stem cells into a liver bud-like tissue using gata6. Nat. Commun..

[88] Kelm J.M., Fussenegger M. (2004). Microscale tissue engineering using gravity-enforced cell assembly. Trends Biotechnol..

[89] Shimizu T., Yamato M., Kikuchi A., Okano T. (2003). Cell sheet engineering for myocardial tissue reconstruction. Biomaterials.

[90] L’Heureux N., Paquet S., Labbe R., Germain L., Auger F.A. (1998). A completely biological tissue-engineered human blood vessel. FASEB J..

[91] Fukuda J., Mizumoto H., Nakazawa K., Kajiwara T., Funatsu K. (2004). Hepatocyte organoid culture in elliptic hollow fibers to develop a hybrid artificial liver. Int. J. Artif. Organs.

[92] Khademhosseini A., Yeh J., Jon S., Eng G., Suh K.Y., Burdick J.A., Langer R. (2004). Molded polyethylene glycol microstructures for capturing cells within microfluidic channels. Lab. Chip.

[93] Putnam A.J., Mooney D.J. (1996). Tissue engineering using synthetic extracellular matrices. Nat. Med..

[94] Marler J.J., Upton J., Langer R., Vacanti J.P. (1998). Transplantation of cells in matrices for tissue regeneration. Adv. Drug Deliv. Rev..

[95] Alberts B., Bray D., Lewis J., Raff M., Roberts K., Watson J.D. (1994). Molecular Biology of the Cell.

[96] Kim B.S., Mooney D.J. (1998). Development of biocompatible synthetic extracellular matrices for tissue engineering. Trends Biotechnol..

[97] Jhon M.S., Andrade J.D. (1973). Water and hydrogels. J. Biomed. Mater. Res..

[98] Zhang Z., He C., Chen X. (2018). Hydrogels based on pH-responsive reversible carbon-nitrogen double-bond linkages for biomedical applications. Master Chem. Front..

[99] Muir V.G., Burdick J.A. (2020). Chemically Modified Biopolymers for the Formation of Biomedical Hydrogels. Chem. Rev..

[100] IUPAC (1996). Glossary of basic terms in polymer science (IUPAC Recommendations 1996). Pure Appl. Chem..

[101] Sperling L.H., Mishra V. (1996). The current status of interpenetrating polymer networks. Polym. Adv. Technol..

[102] Sperling L.H., Klempner D., Sperling L.H., Utracki L.A. (1994). Interpenetrating Polymer Networks.

[103] Sperling L.H., Mark H.F. (2005). Encyclopedia of Polymer Science and Technology.

[104] Ishikawa S., Iijima K., Matsukuma D., Asawa Y., Hoshi K., Osawa S., Otsuka H. (2020). Interpenetrating Polymer Network Hydrogels via a One-Pot and in Situ Gelation System Based on Peptide Self-Assembly and Orthogonal Cross-Linking for Tissue Regeneration. Chem. Mater..

[105] Jensen K.F. (1999). Microchemical systems: Status, challenges, and opportunities. AIChE J..

[106] Stone H.A., Kim S. (2001). Microfluidics: Basic issues, applications, and challenges. AIChE J..

